# YIM-P44. Reduced expression of cd73 on jia synovial lymphocytes is related to cell proliferation

**DOI:** 10.1186/1546-0096-12-S1-Y4

**Published:** 2014-09-17

**Authors:** Sophie Botta Gordon-Smith, Simona Ursu, Robin Callard, Lucy Wedderburn

**Affiliations:** 1 Infection, Inflammation and Rheumatology Section; 2 Immunobiology, UCL Institute of Child Health, London, UK

## Introduction

The nucleoside adenosine exerts regulatory functions prompting T cell anergy and preventing release of inflammatory cytokines. The main source of extracellular adenosine is AMP dephosphorylated by CD73. The resulting product can be taken up by the cell or further metabolized by ADA (Adenosine deaminase) expressed extracellularly when bound to the membrane via coupling to CD26 protein, a surrogate marker for ADA expression. We have shown that CD73 expression and its AMPase activity are reduced on JIA (juvenile idiopathic arthritis) SFMC (synovial fluid mononuclear cells), particularly on CD8^+^ T cells and CD19^+^ B cells; we hypothesise that this may lead to a defect in generation of anti inflammatory adenosine in JIA.

## Objectives

To investigate the mechanisms which lead to reduced cell surface expression of adenosine-generating and degrading ectoenzymes on JIA synovial inflammatory cells

## Methods

PBMC and FACS sorted CD8^+^CD73^+^ from healthy donors were stimulated with anti-CD3mAb and anti-CD28 mAb or CpG, CHO CD40L or anti-IgG/IgM F(ab’)2 and then analysed by flow cytometry for surface markers and the nuclear protein Ki67. In some experiments, cells were labelled with CFSE before culture to allow assessment of proliferation. Culture supernatants were analysed for soluble CD73 protein by ELISA. Data are expressed as medians, analysed by GraphPad Prism.

## Results

Stimulation of T cells through the TCR *in vitro* resulted in reduced CD73 expression on CD8 cells. Higher levels of CD73 protein were found in culture supernatants of TCR stimulated cells (53.8 ng/ml) than that of cells cultured in medium alone (29.9 ng/ml, p=0.03) suggesting that TCR stimulation leads to shedding of CD73 protein. Co-stimulation was not required for this reduction in CD73 expression, as it was still evident after culture with α-CD3 antibody alone. TCR stimulation of sorted CD8^+^CD73^+^ cells demonstrated that CD73 downregulation is related to cell proliferation, as only those cells which had entered cell cycle had lost their CD73 expression. B cell stimulation via use of TLR9 ligand CpG and CHO cells transfected with CD40Ligand led to a significant reduction in CD73 expression on B cells, while culture with anti-IgG/IgM F(ab’)2 induced only a minor, non-significant reduction in CD73 on CD19^+^ cells. Culture in cytokines IL-1, IL-6, TNFα, known to be elevated in the JIA synovium and SF (synovial fluid) did not affect CD73 expression of either T or B cells. Expression of ADA-binding protein CD26 was also found to be reduced on JIA synovial CD8+ T cells (24.7%) compared to both JIA PBMC (63.3%, p < 0.0001) and healthy PBMC (62.9%, p < 0.0001). This result was unexpected as CD26 is known to be a marker of activation and JIA SFMC have an activated phenotype.

## Conclusion

These data show that low expression of CD73 on T and B cells in the inflammatory site is related to cell proliferation and shedding of the protein and suggest that diminished CD73 expression on T and B cells found in the joints of children with JIA may have followed stimulation by proliferative signals at the inflamed site.

## Disclosure of interest

None declared

